# Solitary Colonic Ganglioneuroma: A Rare Incidental Finding of Hematochezia

**DOI:** 10.1155/2015/794985

**Published:** 2015-05-14

**Authors:** George Abraham, Sateesh R. Prakash

**Affiliations:** Department of Medicine, Division of Gastroenterology and Hepatobiliary Disease, New York Methodist Hospital, 506 6th Street, Brooklyn, NY 11215, USA

## Abstract

Ganglioneuromas (GNs) are hamartomatous tumors derived from the autonomic nervous system. It is rare to encounter GN in the gastrointestinal tract. Patients with these tumors usually present with abdominal pain, constipation, ileus, weight loss, or even bleeding. GNs are categorized into three different morphological subtypes, namely, polypoid GN, ganglioneuromatous polyposis, and diffuse ganglioneuromatosis. We present a case of hematochezia from GN in a colon polyp discovered on diagnostic colonoscopy. Due to a lack of guidelines, we reviewed the literature to discuss treatment and other associated conditions for GN.

## 1. Introduction

GNs are uncommon, especially in the gastrointestinal tract. Patients with GN can present with constipation, obstruction, abdominal pain, weight loss, and bleeding depending on the lesions size and location. We present a case of hematochezia from GN in a colon polyp discovered on diagnostic colonoscopy.

## 2. Case Presentation

**Figure 1:**
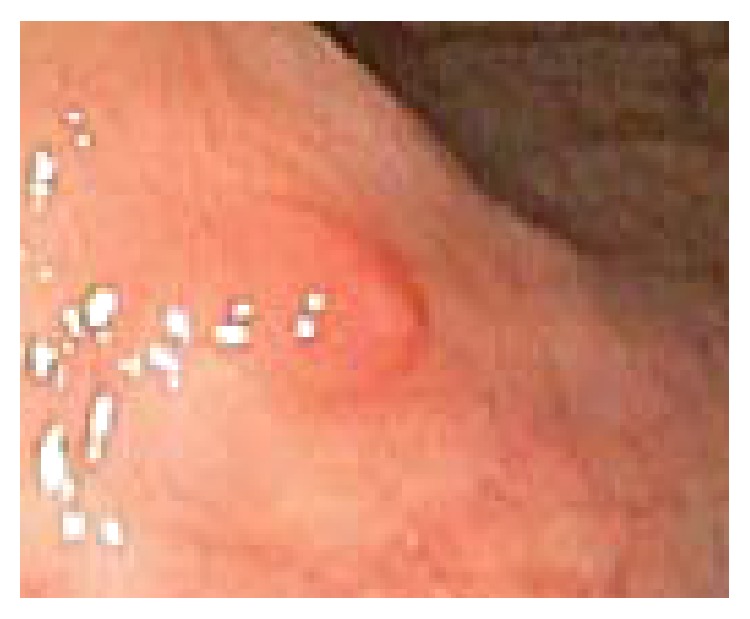
Endoscopic image of the 0.6 cm sessile polyp found in the cecum.

A 43-year-old male with medical history of asthma presents with self-limited episode of hematochezia for 2 days. The patient denies any melena, hematemesis, abdominal pain, constipation, diarrhea, weight loss, or NSAID use. The vital signs and physical examination were within normal range. Routine labs including complete blood count, comprehensive metabolic panel, and coagulation profile were normal. A diagnostic colonoscopy was then performed which revealed a 0.6 cm sessile polyp in the cecum (see Figure 1). A hot forceps polypectomy was performed and routine immunostaining revealed S100 positivity. Histologic examination revealed a ganglioneuroma.

## 3. Discussion

The incidence of lower gastrointestinal bleeding in the United States is estimated to be approximately 36/100,000 cases [1]. Bleeding resulting from intestinal ganglioneuromas is very rare. Of the total of 15 case reports regarding intestinal ganglioneuromas, only 4 presented with bleeding. Intestinal ganglioneuromas often have no symptoms and are usually found incidentally on routine colonoscopic screening.

Ganglioneuromas (GNs) are hamartomatous tumors derived from the autonomic nervous system. These tumors rarely present in the gastrointestinal tract and most frequently occur in head, neck, or adrenal glands. They typically originate from the undifferentiated neural crest, including ganglion cells, nerve fibers, and supporting cells of the enteric nervous system (e.g., glial cells) [2]. They are categorized into three different morphological subtypes, namely, polypoid GNs, ganglioneuromatous polyposis, and diffuse ganglioneuromatosis [3]. Polypoid GNs are typically small (≤2 cm) juvenile polyps that are either adenomatous or hyperplastic. Morphologically, they can be sessile or pedunculated and are often solitary or found in small groups. Ganglioneuromatous polyposis usually has many polyps (often 20 or more) that can be sessile or pedunculated and present in the mucosa and/or submucosa, ranging in size from 1 mm to 2.2 cm. These polyps can be histologically indistinguishable from polypoid GN or may be filiform projections that contain ganglion cells, absent of other neural tissues. Diffuse ganglioneuromatosis is nodular and diffuse tissue that is either transmural or mucosal and involves the myenteric plexus. These lesions can be much larger, ranging up to 17 cm. Histologically, they are either fusiform, hyperplastic projections, or irregular transmural proliferations that are confluent with the myenteric plexus. Adults tend to have mucosal lesions while children can have both mucosal or transmural lesions [4]. Ganglioneuromatous polyposis and diffuse ganglioneuromatosis typically occur in association with neurofibromatosis 1 (also known as von Recklinghausen's disease) or multiple endocrine neoplasia 2B syndrome (MEN 2B), juvenile polyposis, and nonfamilial adenomatous polyposis. Cowden's disease and Ruvalcaba-Myhre-Smith syndrome are more commonly associated with ganglioneuromatous polyposis compared to the other types. Isolated GNs are not typically associated with genetic syndromes [5].

**Figure 2:**
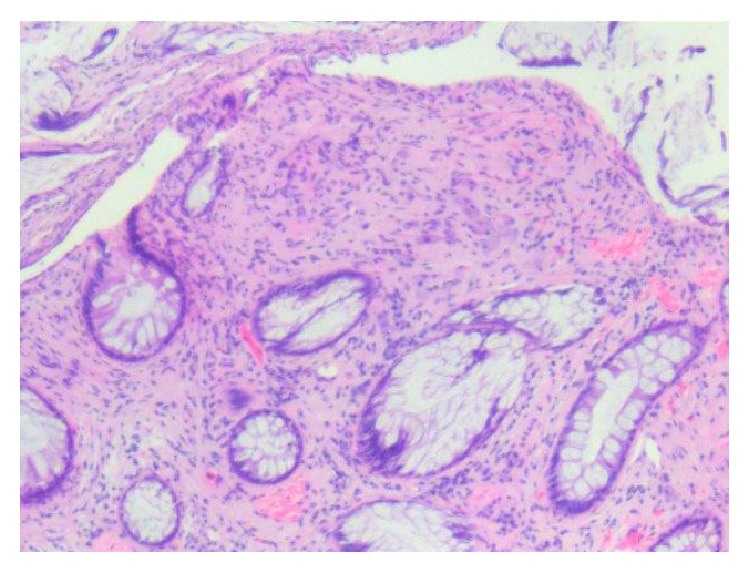
Hematoxylin and eosin stain of the colonic ganglioneuroma. Ganglion and stromal cells are present in the lamina propria. Magnification: 4x.

**Figure 3:**
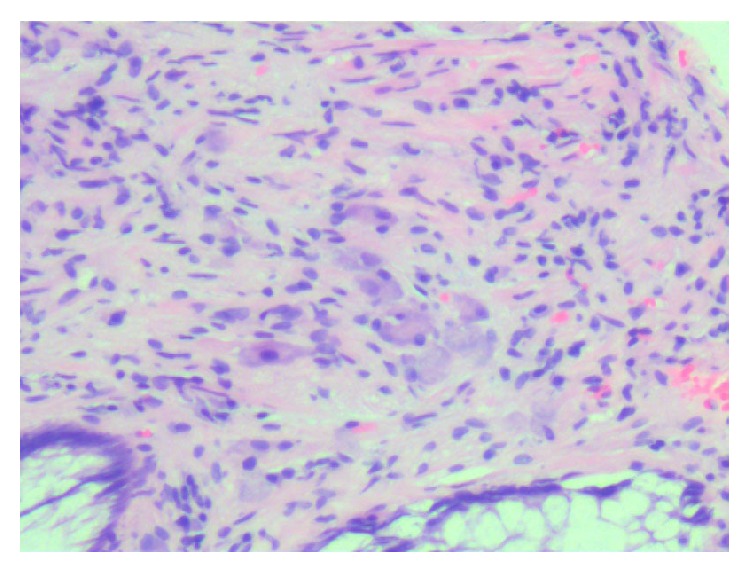
Hematoxylin and eosin stain of the colonic ganglioneuroma. Ganglion and stromal cells are present in the lamina propria. Magnification: 100x.

**Figure 4:**
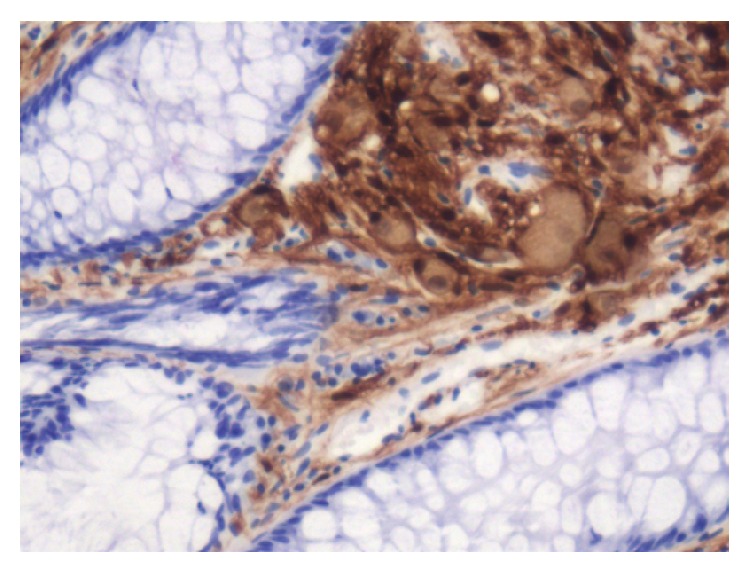
Immunohistochemical stain of the colonic ganglioneuroma demonstrating S100 immunoreactivity. Magnification: 100x.

Patients with solitary GN are often asymptomatic but can present with constipation, abdominal pain, weight loss, obstruction, ileus, and bleeding depending on the lesions size and location. Treatment of solitary GN involves endoscopic resection via hot biopsy forceps. Histology of the lesion will show multiple spindle cells on hematoxylin and eosin stain and will be immunoreactive to S100 protein stain and neuron-specific enolase (see Figures 2, 3, and 4). Currently, no consensus recommendation exists on management of polypoid GN; however, it would be beneficial to schedule a follow-up surveillance colonoscopy to ensure complete excision of the lesion. A few sparse cases of colon cancer coexisting with ganglioneuromatous polyposis or diffuse ganglioneuromatosis have been reported [6–8]. However, there is a lack of data on the association of polypoid GN and colon cancer. Patients should be screened for other associated genetic syndromes and for tumors in other sites like the thyroid, colon, breast, and uterus [9]. Urine vanillylmandelic acid, serum calcitonin, and serum calcium should also be performed to exclude MEN 2B. Our patient had an endoscopic ultrasound with colonoscopy to reevaluate margins of excision, which did not reveal any residual tissue.

In conclusion, GN is a rare cause of hematochezia in adults and confirmatory diagnosis can only be revealed through histologic examination. Further tests for systemic and familial diseases should be considered in patients with GN.
